# Contrast-enhanced endoscopic ultrasound for vascular mapping: a paradigm shift from target enhancement to danger marking in endoscopic ultrasound-guided biliary drainage

**DOI:** 10.1055/a-2952-8509

**Published:** 2026-09-17

**Authors:** Shan-Shan Hu, Wei-Hui Liu

**Affiliations:** 1 Department of Gastroenterology and HepatologySichuan Provincial People's Hospital, School of Medicine 89669 University of Electronic Science and Technology of ChinaChengduSichuan ProvinceChina

Endoscopic ultrasound–guided biliary drainage (EUS-BD) requires precise puncture while avoiding vascular structures. Color Doppler is the standard method for identifying vascular structures, but various causes can produce motion artifacts, resulting in blurred or weakened blood flow signals and creating a false “safe zone.” 1​ 2​ 3​ 4​ ​5​ This report describes a case in which contrast-enhanced endoscopic ultrasound (CE-EUS) was used for vascular mapping after color Doppler imaging was interfered with cardiac pulsation, followed by successful EUS-BD.

A 60-year-old male patient with obstructive jaundice underwent EUS-BD. EUS scanning revealed an anechoic tubular structure. Color Doppler imaging showed intermittent color signals in this structure – sometimes with color, sometimes without – with unclear and flickering blood flow signals ( Fig. 1 ). The structure could not be determined to be a vessel or an intrahepatic bile duct, posing an extremely high risk of erroneous puncture. To clarify the anatomical relationship, 2.5 mL of sulfur hexafluoride microbubbles (SonoVue) was injected intravenously. Contrast-enhanced imaging clearly demonstrated the structure as a vascular channel with clear course and caliber ( **Figs.** 2 and 3 ). Under the guidance of the contrast-marked vascular course, a 19-gauge FNA needle was used to avoid the contrast-filled vessel and puncture the adjacent non-enhanced bile duct ( Fig. 4 ). Cholangiography confirmed successful biliary access. A 0.025-inch guidewire was advanced into the bile duct, followed by the tract dilation and placement of a nasobiliary drainage tube, successfully achieving biliary drainage ( Fig. 5 ). No bleeding, perforation, or other complications occurred during the procedure. Postoperative bilirubin levels normalized within 5 days ( Video 1 ).

**Fig. 1:**
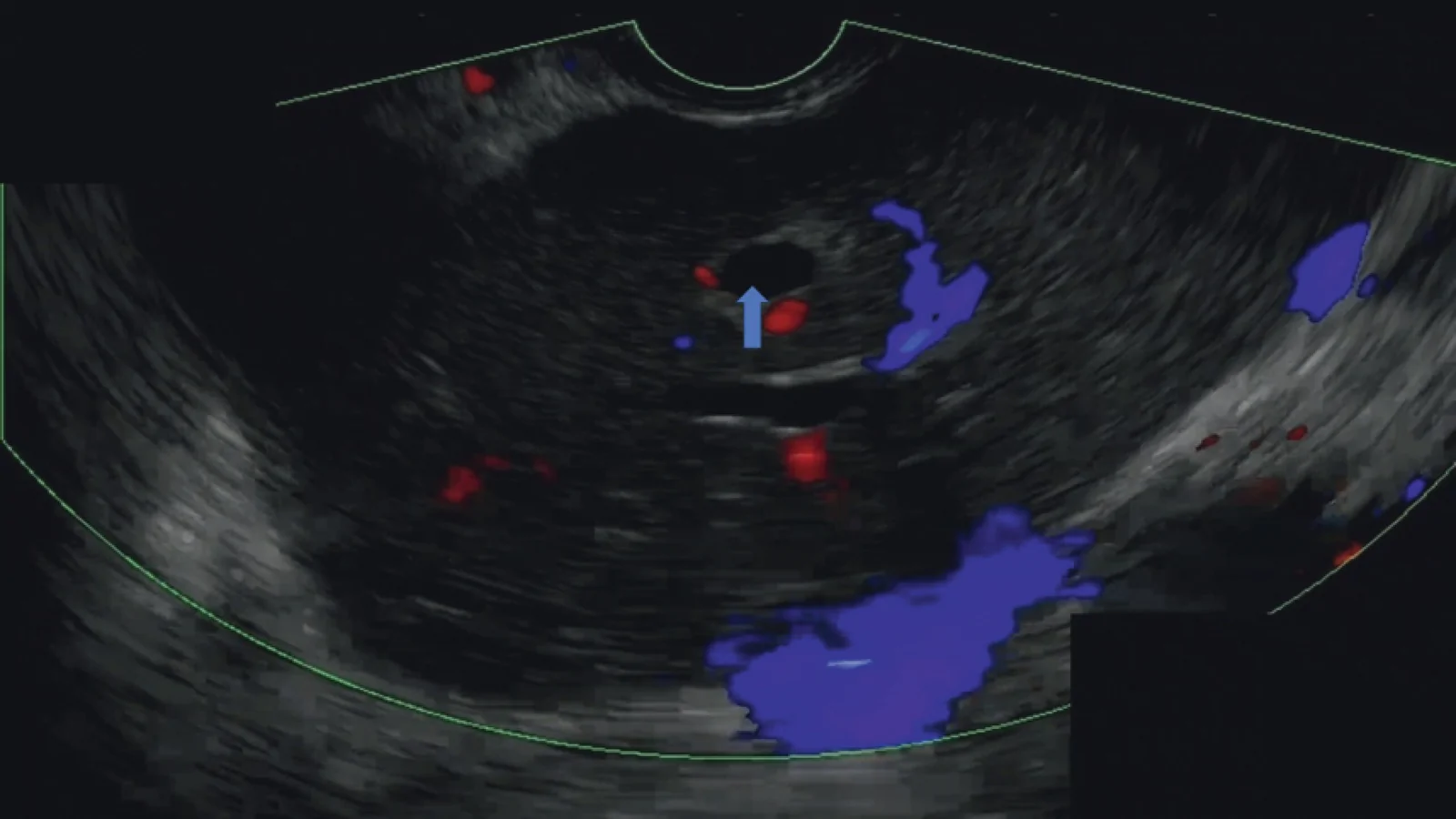
The intermittent and flickering color signals create an unreliable pattern, making it impossible to definitively distinguish between a vessel and an intrahepatic bile duct (blue arrow).

**Fig. 2:**
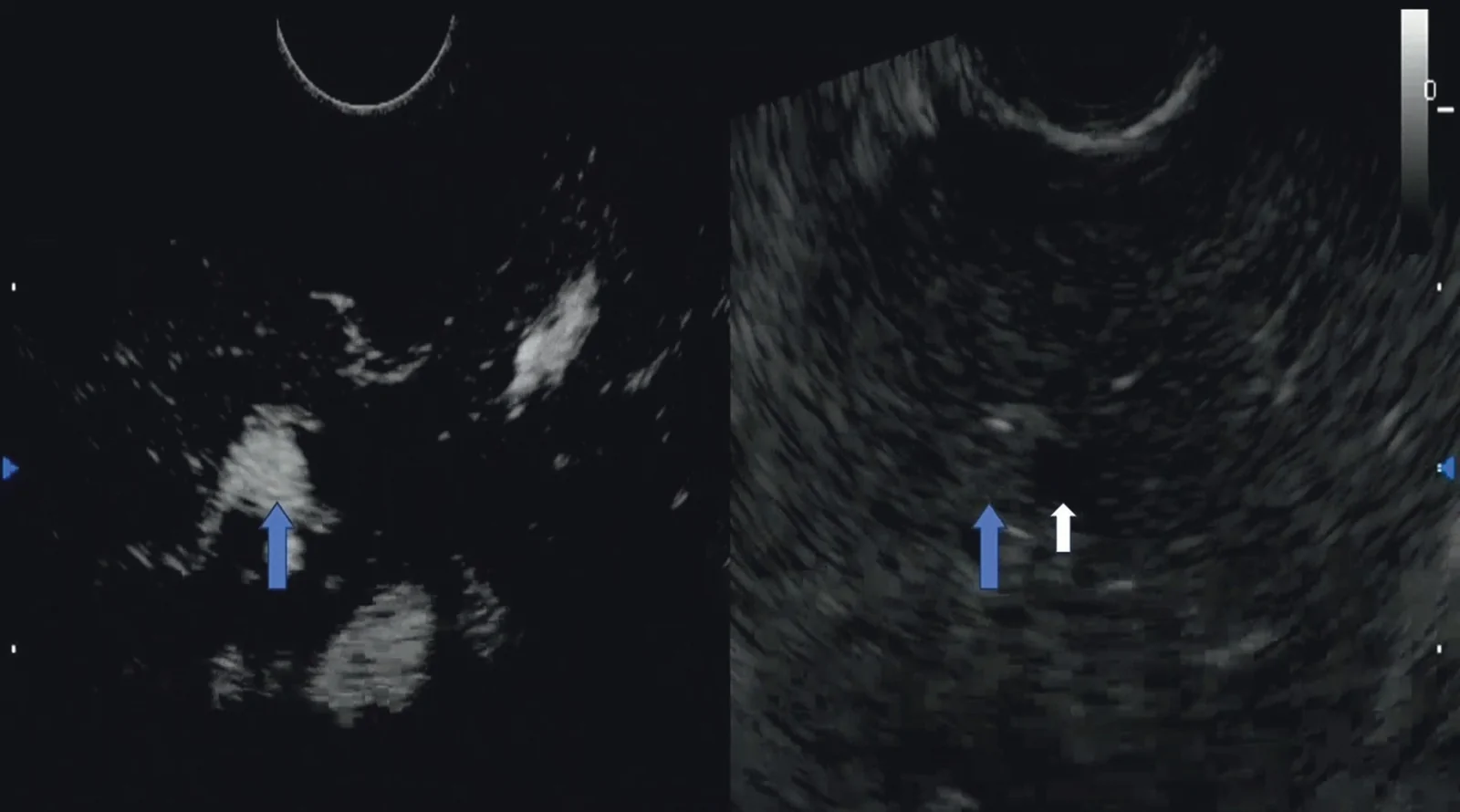
CE-EUS enabling definitive vascular identification. (Left) After intravenous injection of 2.5 mL sulfur hexafluoride microbubbles (SonoVue), the previously ambiguous structure shows bright, homogeneous microbubble enhancement, confirming it as a vascular channel (blue arrow). The vessel course and caliber are clearly delineated. (Right) The corresponding B-mode image showing the same vascular structure (blue arrow) as an anechoic tubular structure adjacent to the non-enhanced bile duct (white arrow).

**Fig. 3:**
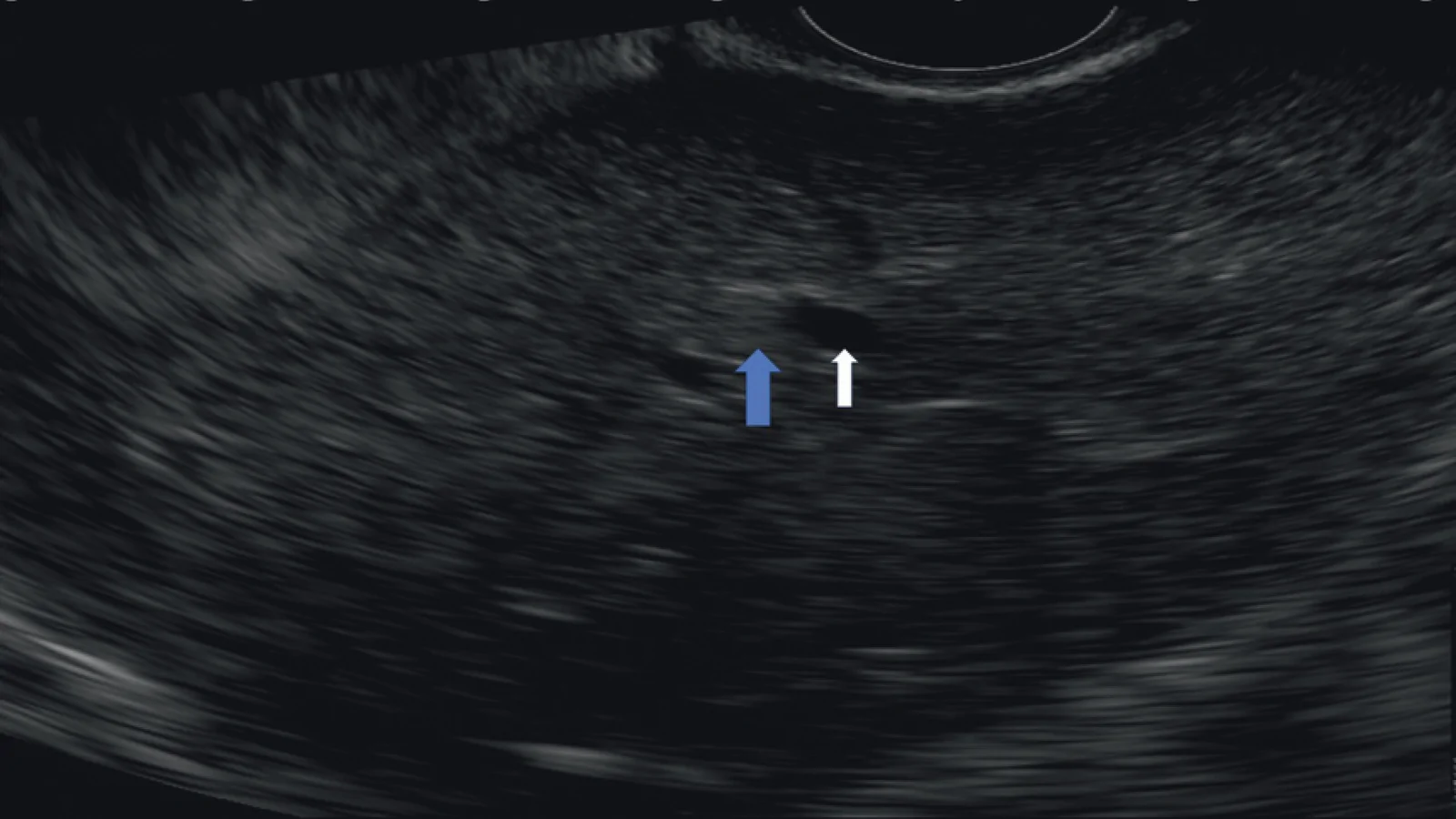
The contrast-enhanced vascular structure (blue arrow) and the non-enhanced bile duct structure (white arrow).

**Fig. 4:**
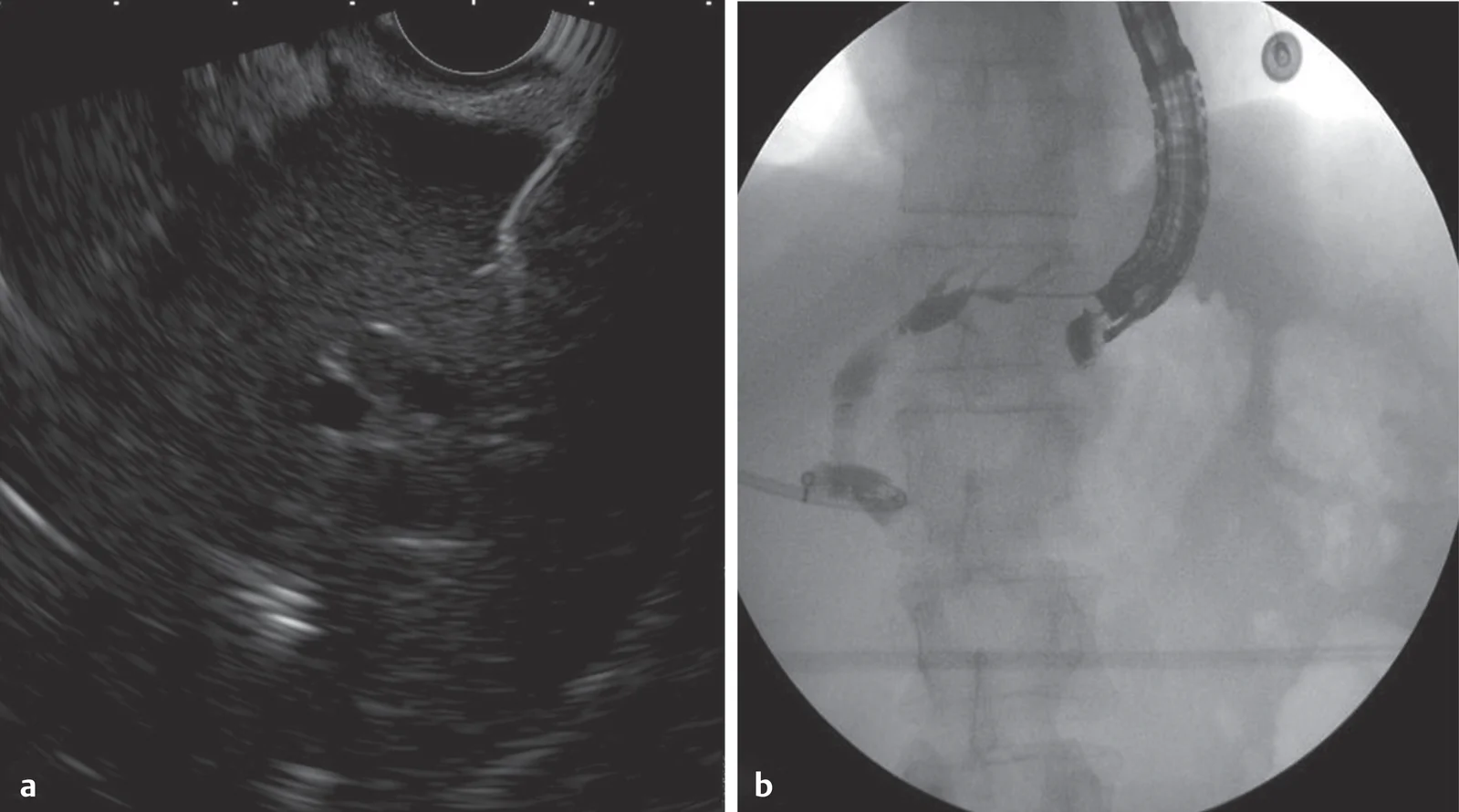
Real-time EUS-guided needle puncture with vascular avoidance. ( **a** ) Under CE-EUS guidance, the contrast-filled vessel is clearly identified and mentally mapped. The 19-gauge FNA needle (white linear echo) is advanced into the adjacent non-enhanced intrahepatic bile duct, deliberately maintaining a safe distance from the vascular structure. The precise needle trajectory demonstrates the practical application of the “danger marking” strategy. ( **b** ) Cholangiography confirms successful biliary access.

**Fig. 5:**
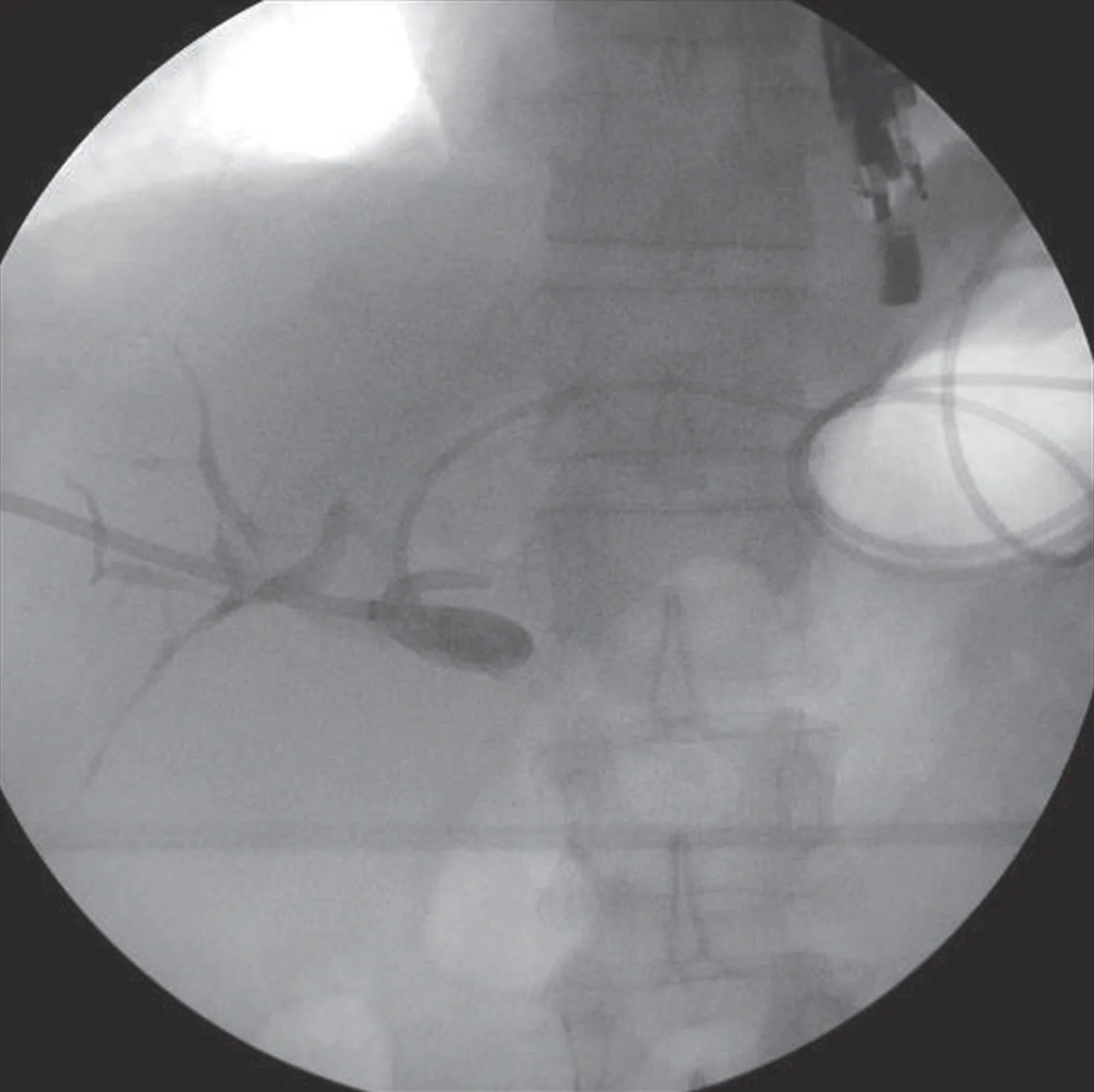
A fluoroscopic image showing successful biliary drainage with a nasobiliary drainage tube in place.

**Video 1** Contrast-enhanced endoscopic ultrasound for vascular mapping: A paradigm shift from target enhancement to danger marking in endoscopic ultrasound–guided biliary drainage.


This case illustrates two key technical points: First, cardiac pulsation and other causes can render color Doppler ineffective during EUS-BD. Second, CE-EUS can be transformed from “target enhancement” to “danger marking”—a reverse targeting strategy in which contrast is used to define structures to be avoided rather than structures to be entered. This paradigm shift is particularly important when conventional Doppler fails. Vascular injury is a serious complication of EUS-BD. Previous reports have mostly described CE-EUS for biliopancreatic duct visualization, but its application for actively excluding vessels in pulsation-interference scenarios has not been emphasized. This case suggests that CE-EUS should be used as a routine pre-procedural vascular mapping tool rather than merely a rescue measure, which is expected to reduce bleeding risk and improve the safety of EUS-BD.

Endoscopy_UCTN_Code_TTT_1AS_2AD

## Declaration of generative artificial intelligence use

The authors confirm that no generative artificial intelligence was used in the preparation of this manuscript.
